# Retinal vascular development in an immature retina at 33–34 weeks postmenstrual age predicts retinopathy of prematurity

**DOI:** 10.1038/s41598-020-75151-0

**Published:** 2020-10-22

**Authors:** Ji Hye Jang, Yu Cheol Kim

**Affiliations:** Department of Ophthalmology, Keimyung University School of Medicine, 1095 Dalgebeol-daro, Dalseo-gu, Daegu, 42601 Republic of Korea

**Keywords:** Health care, Medical research

## Abstract

In preterm birth, the immature retina can develop a potentially blinding disorder of the eye known as retinopathy of prematurity (ROP). The vaso-proliferative phase of ROP begins at an approximate postmenstrual age (PMA) of 32 weeks. There is little or no evidence of an association between ROP development and retinal status in the early vaso-proliferative phase. We aimed to evaluate the retinal vascular findings of infants at 33–34 weeks PMA to determine their risk of ROP. We reviewed 130 serial wide-field retinal images from 65 preterm infants born before the gestational age of 31 weeks. ROP occurred more frequently in infants having a leading vascular edge within posterior Zone II. This was in contrast to normal infants, who are characterized by complete retinal vascularization up to Zone II at 34 weeks PMA. The probability of ROP development in preterm infants with retinal edge hemorrhage was 24.58 times higher than in preterm infants without retinal edge hemorrhage. Eyes with ROP that required treatment showed significantly delayed retinal vascularization accompanied by pre-plus disease. In conclusion, retinal status in the early vaso-proliferation phase might determine the risk of ROP.

## Introduction

Retinopathy of prematurity (ROP) is a fibrovascular proliferative disease affecting premature infants. It is characterized by an arrest or disruption of normal retinal vascular development^1,2^. The retinal blood vessels grow from the optic nerve, traverse the inner retina at about 16 weeks of gestation, and eventually reach the temporal peripheral retina at approximately 40 weeks of gestational age (GA)^3^. Thus, preterm infants have incomplete vascularization of their peripheral retinal area at birth. Therefore, the complete development of their retinal capillary networks occurs after birth^3^.

ROP has two phases: the vaso-obliteration phase and the vaso-proliferation phase^1–4^. In the vaso-obliteration phase (22–32 weeks of gestation), the relative hyperoxic state and insufficient serum insulin-like growth factors levels to which preterm infants are exposed contribute to the suppression of new capillary formation. The vaso-proliferation phase of ROP begins at 32–34 weeks of gestation and is characterized by the formation of new abnormal vessels caused by the overexpression of angiogenic factors due to peripheral retinal hypoxia^3,5^.

In the United states, an initial ROP screening is recommended at 31 weeks postmenstrual age (PMA) or 4 weeks after birth, depending on the time of birth^6^. We currently perform the screening examination for ROP using an indirect ophthalmoscope and various retinal imaging tools. Digital retinal imaging, such as wide-field fundus photography, fluorescein angiography, and spectral-domain optical coherence tomography, help detect subclinical changes, record the retinal status, and facilitate remote image interpretation^7–9^.

It is questionable whether conducting a retinal examination before the recommended time is helpful in the early detection of ROP. When infants are born before 27 weeks GA, and fundus examination is performed at or before 30 weeks PMA, it is often impossible to assess the retinal status because of corneal clouding or the presence of remnants of the tunica vasculosa lentis^10^. Corneal edema is one of the major causes of poor image quality during assessment of ROP. The eyelid separation process begins approximately at 20 weeks GA, and most infants can blink at 31 weeks PMA^11^. Thus, a good retinal image can be obtained after the eyelids are able to open spontaneously and the corneal moisture has dried properly. Therefore, it is suggested that the first eye examination be performed after 31 weeks PMA. The American Academy of Pediatrics guidelines^6^ recommends that the initial ROP examination be performed after this time point. Fortunately, good-quality ROP screening images can be obtained at the beginning of the vaso-proliferation phase.

To date, no study has reported the relationship between early retinal status during the vaso-proliferation phase and the development of ROP. In this study, we analyzed a series of wide-field retinal images obtained from preterm infants and evaluated the relationship between their retinal vascular status at 33–34 weeks PMA and the risk of developing ROP.

## Results

**Figure 1:**
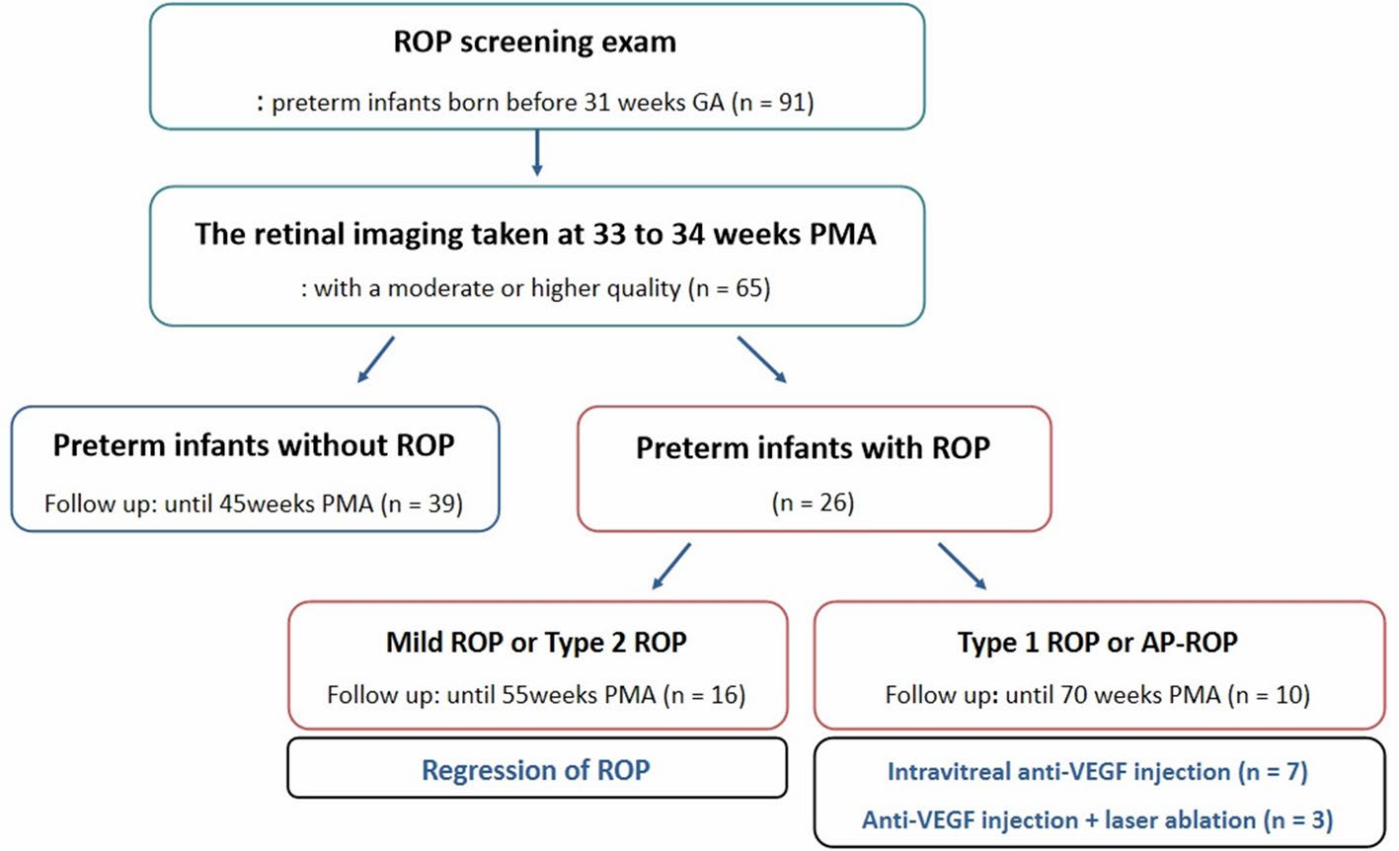
Schematic representation of the data selection procedure followed in this study.

A total of 130 serial wide-field retinal images obtained from 65 preterm infants were included in this study (Fig. 1). The infants and their mothers were of Korean descent. Thirty-nine preterm infants had normal retinal vessel formation that reached the temporal ora serrata, 26 of them developed ROP (any stage), which was diagnosed during a series of follow-up evaluations, and ten infants showed aggressive posterior ROP (AP-ROP) or type 1 ROP, were primarily treated with intravitreal bevacizumab (0.625 mg in 0.025 mL) injections. Of these ten infants, two were later treated with laser ablation, and in one of them, laser ablation and intravitreal injections were administered simultaneously. The demographic characteristics of the preterm infants are shown in Table 1.

**Table 1:** Comparison of preterm infants (n = 65) with and without retinopathy of prematurity.

	Infants without ROP (n = 39)	Infants with ROP (n = 26)	*P*-value
Gender, n (%) male	22 (56.4%)	12 (46.2%)	0.417
**Gestation age at birth (days)**	207 ± 6	195 ± 9	< 0.001*
< 26 weeks, n (%)	0 (0%)	1 (3.8%)	0.400
26 + 0–26 + 6 weeks, n (%)	0 (0%)	6 (23.1%)	0.003*
27 + 0–27 + 6 weeks, n (%)	3 (7.7%)	4 (15.4%)	0.424
28 + 0–28 + 6 weeks, n (%)	8 (20.5%)	10 (38.5%)	0.113
29 + 0–29 + 6 weeks, n (%)	12 (30.8%)	4 (15.4%)	0.158
30 + 0–30 + 6 weeks, n (%)	16 (41.0%)	1 (3.8%)	0.001*
**Weight at birth (g)**	1273 ± 251	1036 ± 230	< 0.001*
500–750 g, n (%)	1 (2.6%)	4 (15.4%)	0.148
751–1000 g, n (%)	6 (15.4%)	9 (34.6%)	0.071
1001–1250 g, n (%)	9 (23.1%)	8 (30.8%)	0.489
1251–1500 g, n (%)	17 (43.6%)	5 (19.2%)	0.042*
> 1500 g, n (%)	6 (15.4%)	0 (0.0%)	0.073

Values of gestation age and weight at birth of each group are presented as mean ± standard deviation.

*ROP* retinopathy of prematurity, *No* number.

*Statistically significant by the Pearson’s chi-square test or Fisher’s exact test.

The retinal findings at 33–34 weeks PMA and the occurrence of ROP are summarized and analyzed in Tables 2 and 3. Approximately 55% of eyes with ROP that required treatment had incomplete retinal vascularization within Zone I at 33–34 weeks PMA. However, all subjects without ROP and those with ROP who did not require treatment revealed complete retinal vessel formation up to Zone I. Complete vascularization up to Zone II was more common in infants without ROP during the ROP workup. Infants with a leading vascular edge within the posterior Zone II area were 9.70 times more likely to develop ROP than those with normal vascularization at 33–34 weeks PMA.

**Table 2:** Summary of retinal findings at 33–34 weeks postmenstrual age, with or without retinopathy of prematurity.

	Without ROP (n = 78 eyes)	With ROP, no treatment required (n = 32 eyes)	With ROP, treatment required (n = 20 eyes)	*P*-value
**Extent of retinal vascularization**				
Complete vascularization up to Zone I, n (%)	78 (100.0%)	32 (100.0%)	9 (45.0%)	< 0.001*
Complete vascularization up to posterior Zone II, n (%)	68 (87.2%)	16 (21.9%)	5 (13.7%)	< 0.001*
**Concomitant vascular abnormalities**				
Demarcation without a line, n (%)	2 (2.6%)	7 (21.9%)	1 (5.0%)	0.128
Retinal edge hemorrhage, n (%)	1 (1.3%)	6 (18.8%)	6 (30.0%)	< 0.001*
Circumferential vessel formation, n (%)	3 (3.8%)	3 (9.4%)	8 (40.0%)	< 0.001*
Pre-plus disease, n (%)	13 (16.7%)	18 (56.2%)	14 (70.0%)	< 0.001*

*ROP* retinopathy of prematurity.

*Statistically significant by the Linear-by-Linear association test.

**Table 3:** Univariate analysis for retinal findings at 33–34 weeks postmenstrual age and the occurrence of retinopathy of prematurity.

Parameters	ROP development risk (130 eyes)			
	Odds ratio	95% CI	Wald *χ*^2^	*P*-value
**Leading vascular edge location**				
Within posterior Zone II area	9.70	4.12–22.84	27.00	< 0.001*
**Concomitant vascular abnormalities**				
Demarcation without a line	6.71	1.38–32.70	5.55	0.019*
Retinal edge hemorrhage	24.58	3.12–193.43	9.25	0.002*
Circumferential vessel formation	7.35	1.97–27.43	8.82	0.003*
Pre-plus disease	8.02	3.53–18.24	24.69	< 0.001*

*ROP* retinopathy of prematurity, *CI* confidence interval.

*Statistically significant by conditional logistic regression analysis.

Vascular abnormalities, such as demarcation without a line, retinal edge hemorrhage, circumferential vessel formation, and pre-plus disease, had significant effects on the occurrence of ROP. Of these, retinal edge hemorrhage had the highest odds ratio, while pre-plus disease revealed the highest Wald χ^2^ value.

The results of a multiple logistic regression analysis were used to identify the predictors for ROP development; they are shown in Table 4. The corresponding areas under the curve (AUC) for the leading vascular edge within posterior Zone II (A) and the presence of pre-plus disease (B) were 0.74 and 0.74, respectively. The AUC for both the parameters (C) was 0.84. The *p*_2_ value was a statistical assessment of the increased value, i.e., the AUC value for each parameter was subtracted from the AUC value of both paremeters. The *p*_2_ values obtained by subtracting the AUC for (A) from (C) and the AUC for (B) from (C) were < 0.001.

**Table 4:** Multiple logistic regression analysis for the risk of developing retinopathy of prematurity based on the location of the leading vascular edge and the presence of pre-plus disease.

Parameter	AUC	95% CI	*P-*value	*P*_2_*-*value
Leading vascular edge within posterior Zone II (A)	0.74	0.66–0.81	< 0.001	
Presence of pre-plus disease (B)	0.74	0.65–0.80	< 0.001	
Both (C)	0.84	0.77–0.90		< 0.001

The *p*_2_ value is a statistical assessment of an increased value, i.e., the AUC value for each parameter is substracted from the AUC value of both parameters.

*ROP* retinopathy of prematurity, *AUC* area under the receiver operating characteristic curve, *CI* confidence interval.

## Discussion

This study aimed to assess the retinal status in the early vaso-proliferation phase and its association with the development of ROP in preterm infants. Three main findings are discussed in this section. First, ROP occurred more frequently among infants with a leading vascular edge within posterior Zone II at 34 weeks PMA than in infants with normal retinal vascularization. Second, ROP was more common in eyes with retinal edge hemorrhage. Third, eyes with ROP that required treatment showed a characteristic delay in retinal vascularization, which was concomitant with pre-plus disease.

Delayed retinal vessel formation up to posterior Zone II at 33–34 weeks PMA is considered a warning sign for ROP occurrence. There exists a negative correlation between ROP occurrence and a low GA or birth weight. However, some preterm infants with extremely low birth weight or those born at an early GA have normal retinal vascularization. Quinn GE et al.^12^ reported that fewer than 10% of eyes without ROP get vascularized into Zone III before 34 weeks PMA, and almost half the eyes (49.4%) get vascularized into Zone III by 37 weeks PMA. When angiogenesis is slow and insufficient in preterm infants, there is a wider avascular peripheral retina at the beginning of the vaso-proliferative phase. As the retina becomes more metabolically active in the vaso-proliferative phase, hypoxia-mediated abnormal angiogenesis occurs in the peripheral ischemic area^2^. In our study, we observed that infants with ROP had incomplete retinal vascularization compared with infants without ROP. In particular, ROP occurred more frequently when retinal vascular growth was incompletely formed up to posterior Zone II at 33–34 weeks PMA.

**Figure 2:**
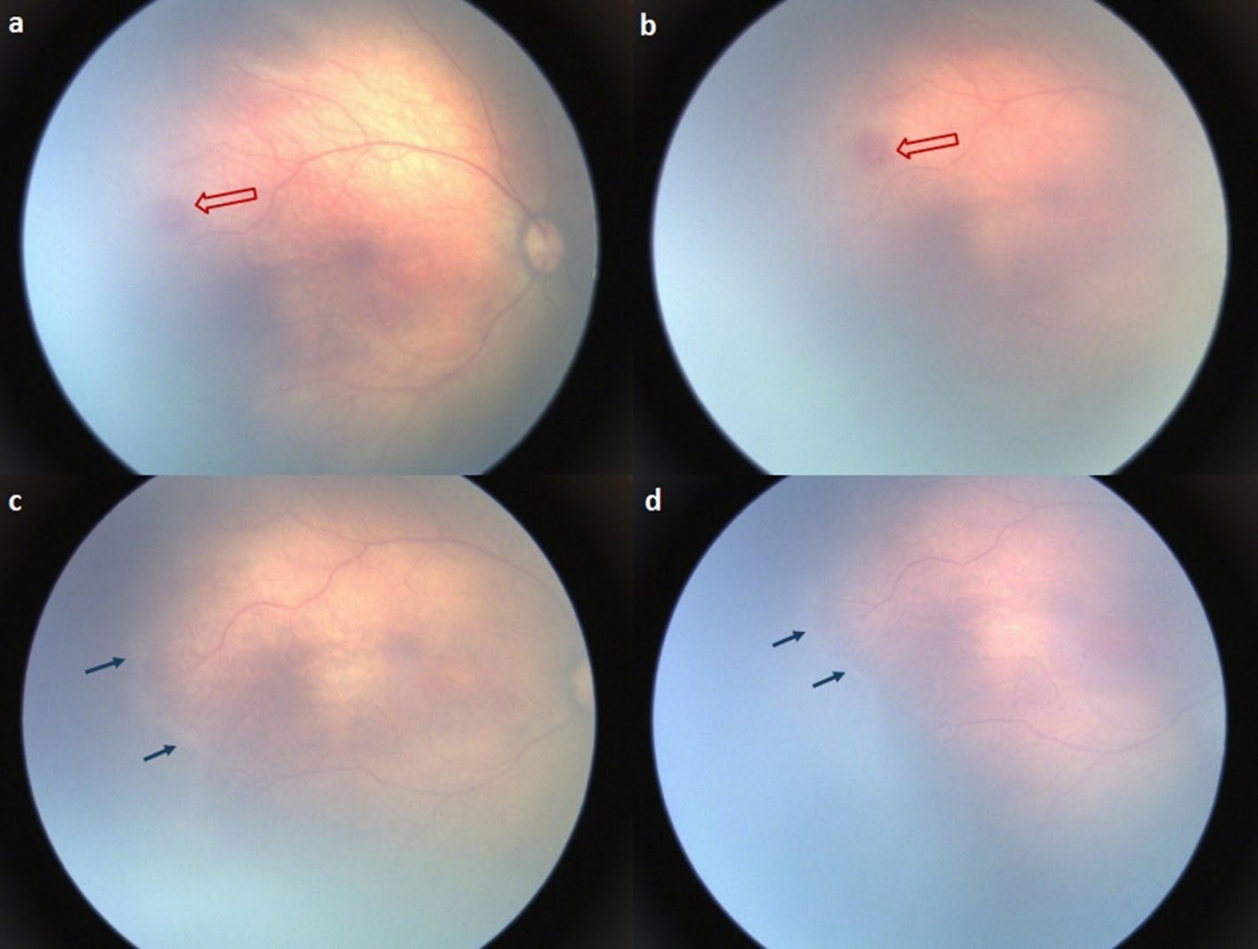
A preterm infant born at 27 + 3 weeks gestational age, with a birth weight of 1190 g. (**a**,**b**) RetCam fundus images of the right eye at 33 + 3 weeks postmenstrual age (PMA), demonstrating a retinal edge hemorrhage (red arrow) at posterior Zone II area. (**c**,**d**) RetCam fundus images at 34 + 3 weeks PMA, demonstrating a demarcation without a line (blue arrows) between stage 0 and stage 1 at the vascular-avascular junction that showed signs of retinal hemorrhage the week before.

In our study, retinal edge hemorrhage, circumferential vessels, and pre-plus disease at 33–34 weeks PMA were more likely to trigger the development of ROP as compared to conditions where they were absent. A remarkable finding was the predictive value for retinal edge hemorrhage; the probability of developing ROP in preterm infants with retinal edge hemorrhage was higher than in those without hemorrhage. Retinal edge hemorrhage appears similar to “Roth’s spots” and is characterized by a red hemorrhagic region on the retina with a white or pale center. The small hemorrhages near the leading vascular edge area (Fig. 2), called “retinal edge hemorrhage,” were observed before the onset of ROP. The hemorrhages mostly disappear within a week or two, although some might persist for several weeks. These hemorrhages frequently occur in conditions prone to damage in retinal capillary endothelial cells.

Changes in oxygen concentration contribute to capillary network remodeling^13^. Oxygen induces apoptosis of the primitive capillary endothelial cells in the retina, thus triggering retinal ischemia. After severe capillary damage, the surviving mature arteries and veins form collateral vessels, or arterio-venous shunts, which are located at the vascular-avascular junction^14,15^. In this study, the retinal edge hemorrhages in eyes with spontaneous regression of ROP appeared similar to those in ROP that required treatment. However, circumferential vessels were more frequently observed in eyes with ROP that required treatment than in ROP with spontaneous regression.

Plus disease is a characteristic sign of ROP progression and acts as a standard marker during its evaluation. However, there is a conflict between the inter- and intra-expert agreement on the assessments of plus disease^16–18^. In addition, the definitions of pre-plus and plus disease are ambiguous. Moreover, its diagnosis depends on the subjective interpretation of the examiner. In our study, the definition of pre-plus disease included the entire range from “dilated or tortuous peripheral branching vessels” to “dilated or tortuous posterior vessels that are insufficient for the diagnosis of plus disease.” ROP eyes that required treatment showed a marked delay in retinal vascularization accompanied by pre-plus disease. We can predict the development of ROP by observing the leading vascular edge within posterior Zone II during the early vaso-proliferative phase, which is concomitant with pre-plus disease. In these cases, a careful follow-up is required.

Our study has several limitations. First, it is a single-center retrospective study. Second, we excluded images that were of a poor quality to enable the evaluation of the leading vascular edge location. This may have led to a selection bias that might have affected the study results. Third, our study included a relatively small number of samples, thereby resulting in a lower statistical power. Fourth, this study might contain errors in expert judgment, because the same retina specialist (J.J.H.) examined the retinal status, determined the ROP diagnosis, and performed the treatment in all patients. Fifth, a broad definition of pre-plus disease was used in this study. Further studies with a more clearly defined definition of pre-plus disease are needed in the future.

In conclusion, the risk for ROP development can be predicted in advance by evaluating the retinal status in the early vaso-proliferation phase. ROP in preterm infants is characterized by delayed retinal angiogenesis, retinal edge hemorrhage, circumferential vessels, and pre-plus disease at 33–34 weeks PMA. In addition, the use of serial wide-field retina images facilitates the assessment of the extent and morphology of retinal vascular formation in preterm infants.

## Methods

This is a single-center retrospective cohort study, conducted between March 2018 and April 2019. We obtained ethical approval from the Institutional Review Board of Keimyung University Hospital (2019-11-072-001) based on the tenets of the Declaration of Helsinki. Written informed consent was obtained from the parents of preterm infants before performing the ROP examination.

### Screening and treatment protocol for retinopathy of prematurity

ROP screening was performed in all infants, born before 31 weeks (up to 30 weeks and 6 days) of gestation, or those having a birth weight of less than 1501 g. The first retinal examination was performed at 31 weeks PMA or 4 weeks after birth, based on the GA at birth.

During their admission, retinal examinations were performed by a retina specialist (J.J.H.) in the neonatal intensive care unit (NICU) without sedation and after pupil dilatation. Retinal images were obtained using a 130° wide-angle retinal camera (RetCam: Clarity Inc., Pleasanton, CA, USA) while the vital signs of the infants were continuously monitored. Following the infant's discharge from the NICU, the specialist performed the retinal assessments and obtained images of the eyes during the follow-up visits at the Department of Ophthalmology. Classification and treatment of ROP were based on the guidelines published by the Early Treatment for Retinopathy of Prematurity Cooperative Group^19^ and the revised version of the International Classification of Retinopathy of Prematurity^20^. Follow-up examinations were recommended based on the status of retinal vascular development. Preterm infants without ROP were followed-up until complete retinal vascularization reached the temporal ora serrata or till 45 weeks PMA. Those with mild or type 2 ROP were followed-up until complete regression or till 55 weeks PMA. An intravitreal bevacizumab injection was administered to the infants with AP-ROP or type 1 ROP within 48 h of diagnosis. If persistent or reactivated ROP was observed after administering the injection, peripheral laser ablation was performed under general anesthesia. If there was no ROP reactivation after the injection, follow-up examinations were carried out till at least 70 weeks PMA (Supplementary Table 1).

### Retinal image selection

We reviewed wide-field retinal images obtained from 91 preterm infants captured at 33–34 weeks PMA (from 33 weeks and 0 days to 34 weeks and 6 days). All the infants were born before 31 weeks GA. We selected retinal images (n = 65 infants, 130 eyes) of moderate or high quality that helped us to identify the leading retinal vascular edge location.

**Figure 3:**
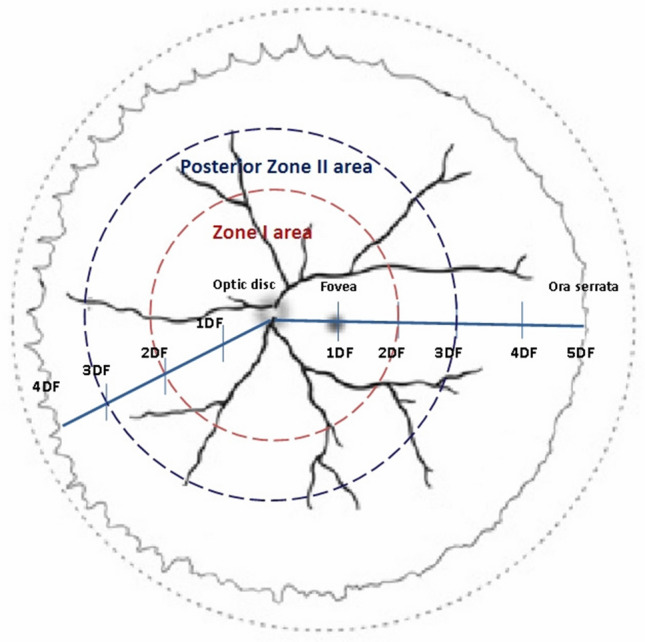
Definition of Zone I area and posterior Zone II area in retinopathy of prematurity. A disc-fovea (DF) unit corresponds to the distance between the center of the optic disc and the fovea center. The Zone I area is defined as the area within a circle of a radius that is twice the DF distance (area inside the red dotted line). The posterior Zone II area is defined as the area inside a circle of a radius that is three times the DF distance (area inside the blue dotted line), excluding the Zone I area.

**Figure 4:**
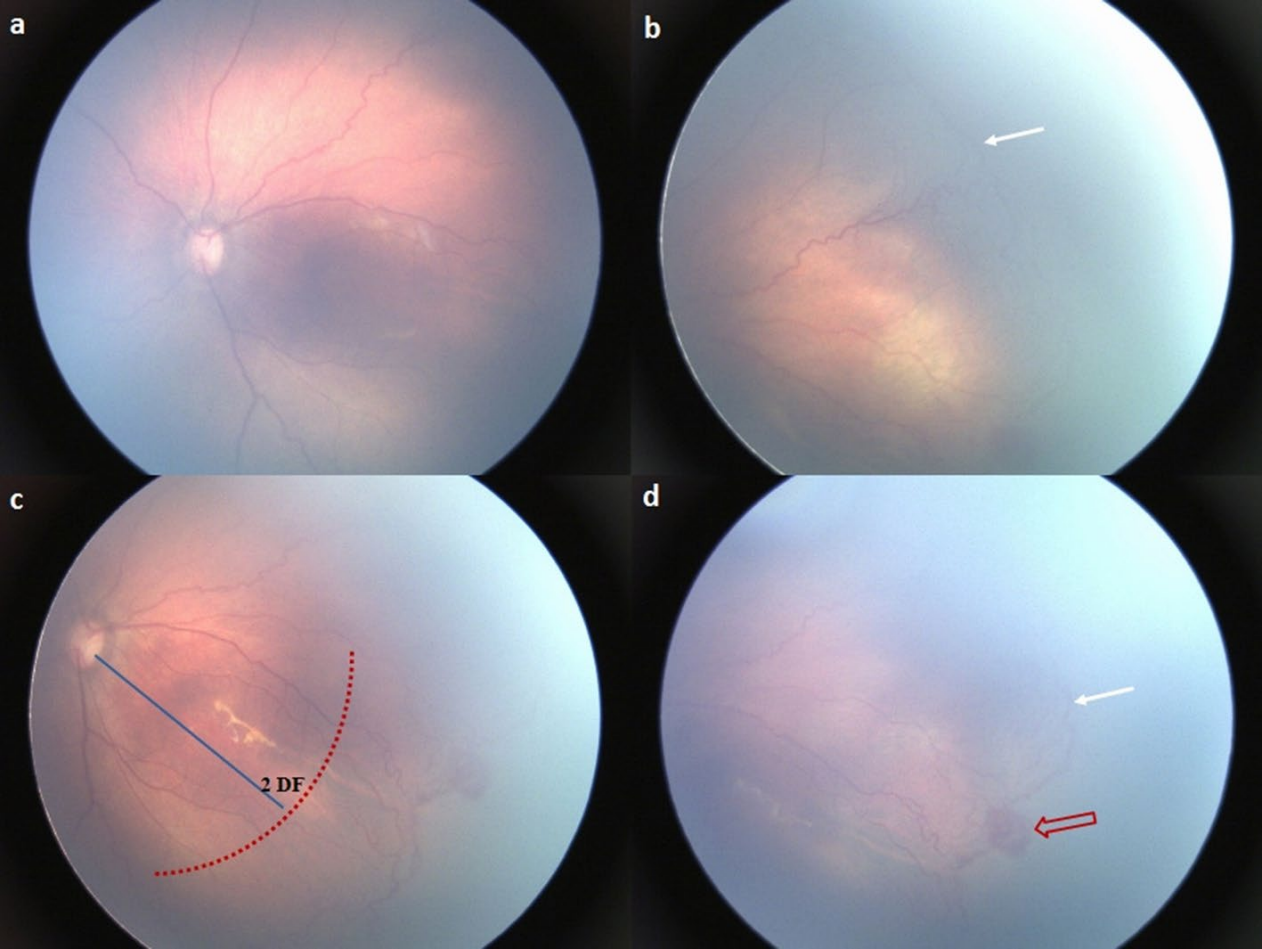
A preterm infant born at 27 + 6 weeks gestational age, with a birth weight of 760 g, progressing into aggressive posterior retinopathy of prematurity. RetCam fundus images of left eye at 34 + 5 weeks postmenstrual age showed the (**a**) pre-plus disease, (**b**) circumferential peripheral vessels (white arrow), (**c**) the leading vascular edge at posterior Zone II (outside the red dotted line) area, and (**d**) a retinal edge hemorrhage (red arrow). The blue line indicates the two disc-fovea (DF) unit.

The extent of retinal vascularization was measured using disc-fovea (DF) units. A DF unit is defined as the distance between the center of the optic disc and that of the fovea. The distance from the center of the optic disc to the nasal ora serrata was measured as 4 DF, while the distance between the center of the optic disc and the temporal ora serrata was measured as 5 DF^21^. The Zone I area was defined as the area within a circle of a radius equal to 2 DF, and the posterior Zone II area was defined similarly as the area with a circle radius of 3 DF, excluding the Zone I area^22^ (Fig. 3). We checked the location of the leading vascular edge at 33–34 weeks PMA. We also assessed early changes, including the following: (1) an indistinguishable white line between stage 0 and stage 1, which was present at the junction of the vascular- avascular retina, and termed “demarcation without a line,” (2) retinal hemorrhage near the leading vascular development area, termed “retinal edge hemorrhage,” (3) formation of circumferential vessels or an arterio-venous shunt inside the vascularized retina, and (4) abnormal vascular morphology ranging from “dilated or tortuous peripheral branching vessels” to “insufficient for plus disease diagnosis,” also referred to as “pre-plus disease” (Fig. 4). The acquired images were analyzed to determine the association between each of the above-mentioned variables and the occurrence of ROP.

### Data analysis

The statistical analysis was performed using the SAS program (version 9.4, SAS Institute Inc. Cary, North Carolina, USA). A value of *p* < 0.05 was considered statistically significant.

The demographic information between infants without ROP and infants with ROP was compared using the Pearson’s chi-square test or Fisher’s exact test. The extent of retinal vascularization and concomitant vascular abnormalities among three groups classified according to the presence and severity of ROP were analyzed using the Linear-by-Linear association test.

Univariate analyses were performed, using odds ratio, 95% confidence interval (CI), and the χ^2^ test to assess the risk factors for ROP. A conditional logistic regression analysis was performed after taking into account the dependence of two eyes in each infant. A multiple logistic regression analysis was conducted for all the significant variables of the univariate analyses (*p* < 0.001). A receiver operating characteristic curve was used to measure the prognostic value of the different variables of ROP development.

## Supplementary information

Supplementary Table 1.
