# Supplementary material for: Retinal vascular development in an immature retina at 33–34 weeks postmenstrual age predicts retinopathy of prematurity
Source: Sci Rep. 2020 Oct 22;10:18111. doi: 10.1038/s41598-020-75151-0 (PMC7582165; doi:10.1038/s41598-020-75151-0)
Supplement: Supplementary file 1 — Supplementary Table 1. [file 41598_2020_75151_MOESM1_ESM.docx]

**Retinal Vascular Development in an Immature Retina at 33-34 Weeks Postmenstrual Age Predicts Retinopathy of Prematurity**

Ji Hye Jang^1,*^and Yu Cheol Kim^1^

^1^Department of Ophthalmology, Keimyung University School of Medicine, Daegu 42601, Republic of Korea

***Corresponding author:** Ji Hye Jang,MD., PhD., Department of Ophthalmology, Keimyung University School of Medicine, 1095 Dalgebeol-daro, Dalseo-gu, Daegu, 42601, Republic of Korea (e-mail: mjmom99@naver.com)

Supplementary table 1. Retinal findings in preterm infants with retinopathy of prematurity that required treatment at 33-34 weeks postmenstrual age and their treatment status according to the protocol

|  | Age at birth (week) | Weight at birth (g) | Eye | Complete vascularization  at 33-34 weeks PMA | | Presence of concomitant vascular abnormality  at 33-34weeks PMA | | | | Occurrence of ROP type | Age at the time of intravitreal anti-VEGF injection  (weeks) | Age at the time of laser ablation  (weeks) | Other characteristics |
| --- | --- | --- | --- | --- | --- | --- | --- | --- | --- | --- | --- | --- | --- |
|  |  |  |  | Zone I | Posterior  Zone II | demarcation without a line | retinal edge hemorrhage | circumferential vessels | pre-plus disease |  |  |  |  |
| Case 1 | 24+6 | 610 | OD | No | No | Yes | No | No | Yes | AP-ROP | 35+3 | 51+0 | Laser ablation due to reactivation of ROP after injection |
|  |  |  | OS | No | No | No | No | Yes | Yes | AP-ROP |  |  |  |
| Case 2 | 26+3 | 850 | OD | No | No | No | No | No | No | AP-ROP | 38+0 | - | Regression of ROP after injection |
|  |  |  | OS | No | No | No | No | No | No | AP-ROP |  |  |  |
| Case 3 | 27+6 | 790 | OD | No | No | No | No | Yes | Yes | AP-ROP | 34+6 | 36+2 | Laser ablation due to no regression of ROP after injection |
|  |  |  | OS | No | No | No | Yes | Yes | Yes | AP-ROP |  |  |  |
| Case 4 | 26+1 | 990 | OD | No | No | No | No | No | No | Type 1 ROP | 36+1 | - | Regression of ROP after injection |
|  |  |  | OS | No | No | No | No | No | No | Type 1 ROP |  |  |  |
| Case 5 | 26+1 | 1050 | OD | Yes | Yes | No | No | Yes | Yes | Type 1 ROP | 48+1 | 48+1 | Simultaneous laser ablation and injection |
|  |  |  | OS | Yes | No | No | No | No | Yes | Type 1 ROP |  |  |  |
| Case 6 | 26+2 | 710 | OD | Yes | Yes | No | No | No | No | Type 1 ROP | 40+3 | - | Regression of ROP after injection |
|  |  |  | OS | Yes | Yes | No | No | No | No | Type 1 ROP |  |  |  |
| Case 7 | 26+4 | 840 | OD | No | No | No | Yes | No | Yes | Type 1 ROP | 37+2 | - | Vitreous hemorrhage after injection, but was absorbed eventually |
|  |  |  | OS | Yes | No | No | No | No | Yes | Type 1 ROP |  |  |  |
| Case 8 | 28+1 | 1040 | OD | Yes | No | No | Yes | Yes | Yes | Type 1 ROP | 39+2 | - | Regression of ROP after injection |
|  |  |  | OS | Yes | No | No | Yes | Yes | Yes | Type 1 ROP |  |  |  |
| Case 9 | 28+1 | 1270 | OD | Yes | Yes | No | Yes | Yes | Yes | Type 1 ROP | 36+5 | - | Regression of ROP after injection |
|  |  |  | OS | Yes | Yes | No | Yes | Yes | Yes | Type 1 ROP |  |  |  |
| Case 10 | 28+4 | 1460 | OD | No | No | No | No | No | Yes | Type 1 ROP | 36+2 | - | Regression of ROP after injection |
|  |  |  | OS | No | No | No | No | No | Yes | Type 1 ROP | 37+2 |  |  |

*PMA*: postmenstrual age; *ROP*: retinopathy of prematurity; *VEGF*: vascular endothelial growth factor; *AP-ROP*: aggressive posterior- retinopathy of prematurity.
